# The Chloroplast Genome of *Symplocarpus renifolius*: A Comparison of Chloroplast Genome Structure in Araceae

**DOI:** 10.3390/genes8110324

**Published:** 2017-11-16

**Authors:** Kyoung Su Choi, Kyu Tae Park, SeonJoo Park

**Affiliations:** Department of Life Sciences, Yeungnam University, Gyeongsan 712-749, Gyeongsangbuk-do, Korea; kschoi01@ynu.ac.kr (K.S.C.); allan57@ynu.ac.kr (K.T.P.)

**Keywords:** Araceae, *Symplocarpus*, *infA*, *psbZ*, inverted repeat region, phylogenetic relationship

## Abstract

*Symplocarpus renifolius* is a member of Araceae family that is extraordinarily diverse in appearance. Previous studies on chloroplast genomes in Araceae were focused on duckweeds (Lemnoideae) and root crops (*Colocasia*, commonly known as taro). Here, we determined the chloroplast genome of *Symplocarpus renifolius* and compared the factors, such as genes and inverted repeat (IR) junctions and performed phylogenetic analysis using other Araceae species. The chloroplast genome of *S. renifolius* is 158,521 bp and includes 113 genes. A comparison among the Araceae chloroplast genomes showed that *infA* in *Lemna*, *Spirodela*, *Wolffiella*, *Wolffia*, *Dieffenbachia* and *Colocasia* has been lost or has become a pseudogene and has only been retained in *Symplocarpus*. In the Araceae chloroplast DNA (cpDNA), *psbZ* is retained. However, *psbZ* duplication occurred in *Wolffia* species and tandem repeats were noted around the duplication regions. A comparison of the IR junction in Araceae species revealed the presence of *ycf1* and *rps15* in the small single copy region, whereas duckweed species contained *ycf1* and *rps15* in the IR region. The phylogenetic analyses of the chloroplast genomes revealed that *Symplocarpus* are a basal group and are sister to the other Araceae species. Consequently, *infA* deletion or pseudogene events in Araceae occurred after the divergence of *Symplocarpus* and aquatic plants (duckweeds) in Araceae and duplication events of *rps15* and *ycf1* occurred in the IR region.

## 1. Introduction

The family Araceae consists of approximately 102 genera and 3300 species and is one of most structurally diverse groups of monocots. This family is widely distributed in all the continents, except Antarctica. The Lemnoideae subfamily (duckweeds) within Araceae displays particularly diverse morphological traits, habits and life forms [1,2,3]. The earliest modern classification of Araceae was given in 1860 by Schott [4] and Engler [5,6]. They classified Araceae according to the vegetable morphology and anatomy. Many molecular phylogenetic analyses support this classification. However, the taxonomic relationships among some species have not been resolved, for example, among the members of Aroideae subfamily [2,3,7,8].

The genus *Symplocarpus* belongs to the subfamily Orontioideae, which also contains the genera *Orontium* and *Lysichiton* [9]. Five *Symplocarpus* species (*S. renifolius* Schott ex Miq., *S. foetidus* (L.) Nutt., *S. nipponicus* Makino, *S. nabekuraensis* Otsika and K.Inoue and *S. egorovii* N.S. Pavlova and V.A. Nechaev) are distributed in eastern Asia and eastern North America [9,10,11].

The plant chloroplast (cp) genomes are divided into four major segments. Two of these contain only single copies of genes: the large single-copy (LSC) region and small single-copy (SSC) region. The last two segments are nearly identical inverted copies, termed inverted repeats (IR_A_ and IR_B_). The cp genomes are approximately 120–170 kb and include 100–120 genes. Most angiosperm cp genomes are highly conserved [12,13]. However, there have been observations of gene loss [14], inversion [15], rearrangement [16], IR loss [17], IR contraction and IR extension [18] in some lineages. In particular, four junctions (LSC/IR_B_, IR_B_/SSC, SSC/IR_A_ and IR_A_/LSC) of cp genome were found at various sites. Wang et al. [18] suggested two possible mechanisms, (i) gene conversion to account for the small IR expansion or movements in most species of the genus; (ii) a DNA double-strand break (DSB) to explain the extensive incorporation of the LSC region. We observed that the IR region has expanded in three species of Lemnoideae.

Previous studies on the cp genomes in the Araceae family have focused on five species. Mardanov et al. [19] studied the cp genome of Duckweed (*Lemna minor)* and compared it with those of the other angiosperm species. Wang and Messing [20] generated the cp genomes of three other duckweed species (*Spirodela polyrhiza*, *Wolffiella lingulata* and *Wolffia australiana*) and Ahmed et al. [21] determined the cp genome sequences for two taro species (subfamily Aroideae) and compared the Aroid cp genomes.

In this study, we determined the complete cp genome sequence of the Asian skunk cabbage *Symplocarpus renifolius* (subfamily Orontioideae, family Araceae) and compared it with those of other Araceae species. We also analyzed the gene contents (*infA* and *psbZ*), IR region extensions (*ycf1* and *rps15* duplication) and phylogenetic relationships in Araceae.

## 2. Materials and Methods

### 2.1. Plants Materials and DNA Sequencing

This study was approved by the Korean National Arboretum (KNA 1-2-13, 14-2). The *S*. *renifolius* sample was obtained from a living collection in the greenhouse of Yeungnam University, in Gyeongsan, South Korea. Total DNA was extracted using a DNeasy Plant Mini Kit (Qiagen Inc., Valencia, CA, USA) and was quantified using a HiGen Gel and PCR Purification system (Biofact Inc., Daejeon, Korea). The genomic DNA was sequenced using Illumina Miseq (Illumina Inc., San Diego, CA, USA). A total number of 4,491,905,669 raw reads were obtained from the Illumina sequencing. These raw reads were trimmed and filtered using Genious v.10.1 (Biomatters, Auckland, New Zealand). The filtered 1,516,987 reads were mapped to reference genome, *Dieffenbachia sequine* (NC_027272). The coverage of *S. renifolia* was 1002 X. The genome coverage was estimated using the CLC Genomics Workbench, v. 7.0.4 (CLC Bio, Aarchus, Denmark) at LabGenomics Co. (Seongnam, Korea).

### 2.2. Genome Annotation, Genome Mapping and Sequence Analysis

The complete cp genome sequence was annotated using Dual Organellar GenoMe Annotator (DOGMA) [22] and was compared with the published complete cp genome sequences (Table S1) available for *Colocasia esculenta* (JN105689), *Dieffenbachia seguine* (NC_0272725), *Spirodela polyrhiza* (JN160603), *Wolffia australiana* (JN160605) and *Wolffiella lingulata* (JN160604). All the identified tRNA genes were verified using the corresponding structures predicted by tRNAscan-SE [23]. A circular cp genome map was drawn using ORDRAW [24]. To compare the structure and genes present in Araceae family, sequences from the different plants were aligned using MAFFT [25] and Geneious v.6.1.7 bioinformatics software platform (Biomatters).

### 2.3. Repeat Structure

Tandem repeats (forward, palindrome, reverse and complement repeats) were detected using REPtuer program [26]. The minimum repeat size was set at 30 bp and at a sequence identity greater than 90%. The simple sequence repeats (SSRs) were detected using Phobos v.3.3.12 (http://www.ruhr-uni-bochum.de/ecoevo/cm/com_phobos.htm). We applied a threshold 10 to mononucleotide repeats, five to dinucleotide repeats, four to trinucleotide repeats, three to tetranucleotide repeats and two to penta-, hexa-, hepta- and deca- nucleotide repeats.

### 2.4. Phylogenetic Analysis

A total of 77 coding genes from 17 species were compiled into a single file of 67,392 bp and aligned using MAFFT [25]. Sixteen species (including six Araceae species) were selected as the in-groups and *Amborella trichopoda* was included as the out-group (Table S1). Maximum likelihood (ML) analyses were performed using RAxmL v.7.4.2 with 1000 bootstrap replications and using the GTR+I+G model [27]. The best-fit models of substitutions selected by ModelTest 3.7 [28].

## 3. Results

### 3.1. Characteristics of the Symplocarpus renifolius and Araceae cp Genomes

**Figure 1:**
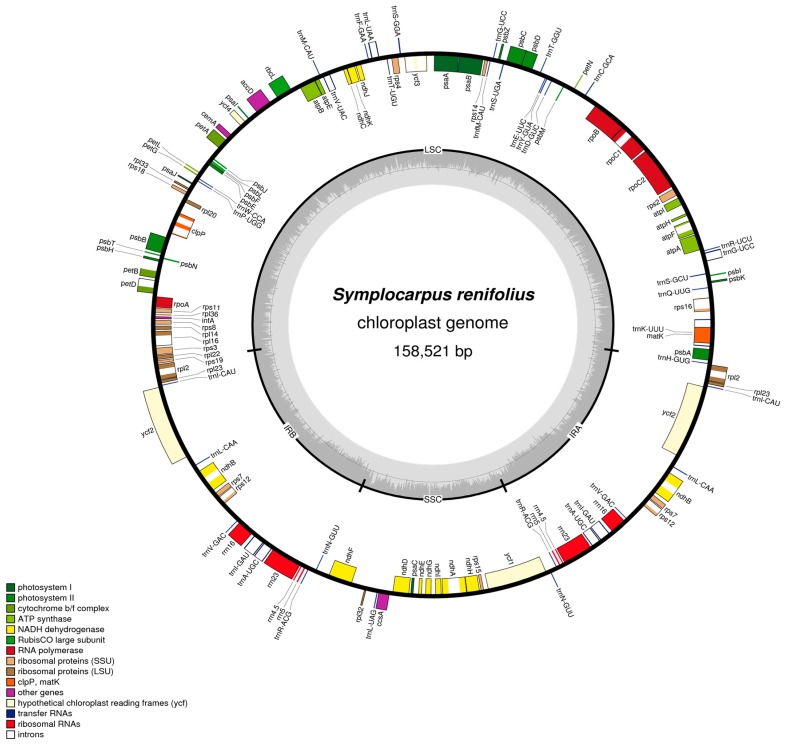
Complete chloroplast genome of *Symplocarpus renifolius*. Genes drawn inside the circle are transcribed clockwise, whereas those outside are transcribed counterclockwise. The gray plot in the inner circle corresponds to the GC content. The colored bars indicate known protein-coding genes, transfer RNA (tRNA) genes and ribosomal RNA (rRNA) genes.

The cp genome of *S. renifolius* (GenBank accession number KY039276) is 158,521 bp in length (Figure 1). It includes two inverted repeat regions (IR_A_ and IR_B_) of 25,801 bp separated by LSC and SSC regions of 86,620 bp and 20,299 bp, respectively. The overall AT content of the *S. renifolius* cp genome is 62.7% (Table 1). The *S. renifolius* cp genome contained 113 genes, of which four were ribosomal RNA (rRNA) genes, 30 were transfer RNA (tRNA) genes, 78 genes were protein coding genes and 17 genes located in the IR regions were duplicated (*rpl2*, *rpl23*, *trnI*-CAU, *ycf2*, *trnL*-CAA, *ndhB*, *rps7*, *rps12*, *trnV*-GAC, rrn16, *trnI*-GAU, *trnA*-UGC, rrn23, rrn4.5, rrn5, *trnR*-ACG and *trnN*-UGA). Of the 113 single-copy genes, 13 contained a single intron (eight protein-coding genes and five tRNA genes) and three protein-coding genes (*ycf3*, *clpP* and *rps12*) contained two introns.

The seven complete cp genomes of Araceae species (*Lemna*, *Spirodela*, *Wolffiella*, *Wolffia*, *Dieffenbachia*, *Colocasia* and *Symplocarpus*) consist of a pair of IRs (25,273–31,930 bp) separated by one LSC (86,670–92,015 bp) and one SSC (13,394–22,208 bp) region, each. *S. renifolius* has one of the smallest cp genomes and has the lowest AT content among the Araceae species (Table 1).

### 3.2. infA and psbZ Genes in Araceae

**Figure 2:**
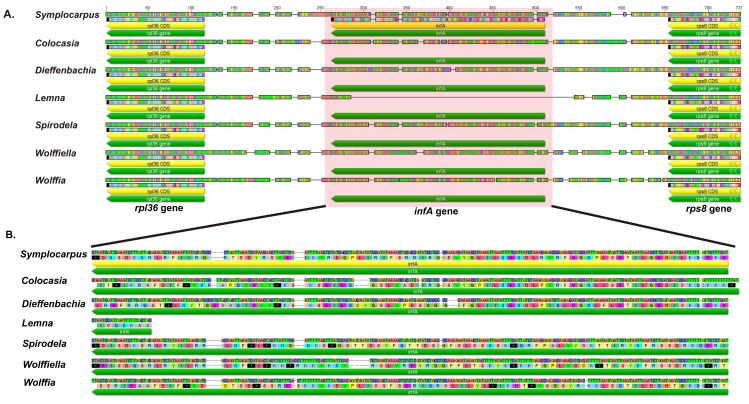
Sequence alignment of the *rpl*36*-rps*8 region (including *infA*) in seven Araceae species. (**A**) *rpl36*-*rps8* region; (**B**) *infA* sequence and its translation.

In the present study, we compared each individual genes, rearrangement and IR boundaries in the cp genomes of *Symplocarpus* and other species of Araceae. The genes are conserved, except for *infA* and *psbZ*, within Araceae. Previous study suggested that the functional gene, *infA* is highly variable in Araceae species such as pseudogene or missing gene [21]. However, *infA* of *S. renifolius* is an intact gene (Figure 2).

**Figure 3:**
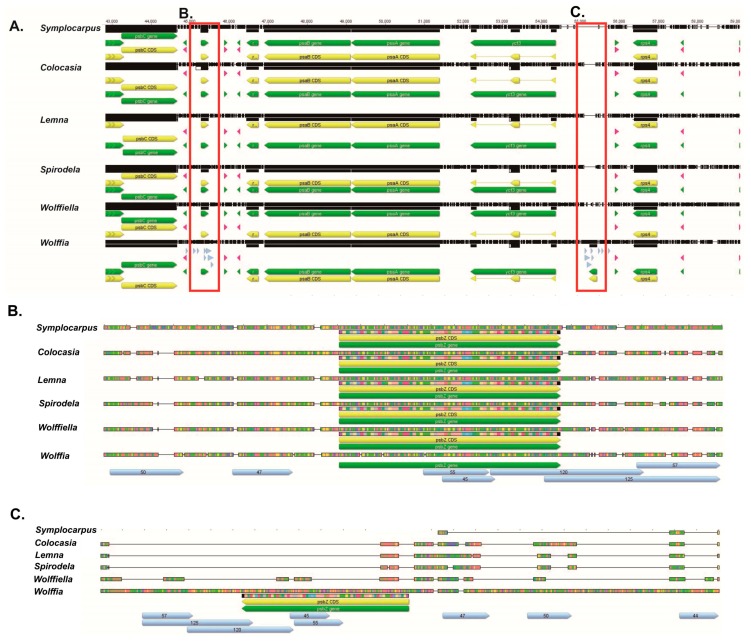
Comparison of *psbZ* in Araceae species. (**A**) Sequence of *psbC*-*rps4* region; (**B**) Location of *psbZ* between *trnS*-UGA and *trnG*-UCC (**C**) Location of *psbZ* between *ycf3* and *trnS*-GGA. Arrow indicates the tandem repeats.

*psbZ* in six Araceae species (*Lemna*, *Spirodela*, *Dieffenbachia*, *Wolffiella*, *Colocasia* and *Symplocarpus*) was located between *trnS*-UGA and *trnG*-UCC. However, *psbZ* of *Wolffia* had a duplication event because of which two *psbZ* genes became one pseudogene and one intact gene. The *psbZ* pseudogene was located between *trnS*-UGA and *trnG*-UCC and the intact *psbZ* was located between *ycf3* and *trnS*-GGA (Figure 3).

### 3.3. Simple-Sequence Repeats Loci of Araceae cp Genomes

**Figure 4:**
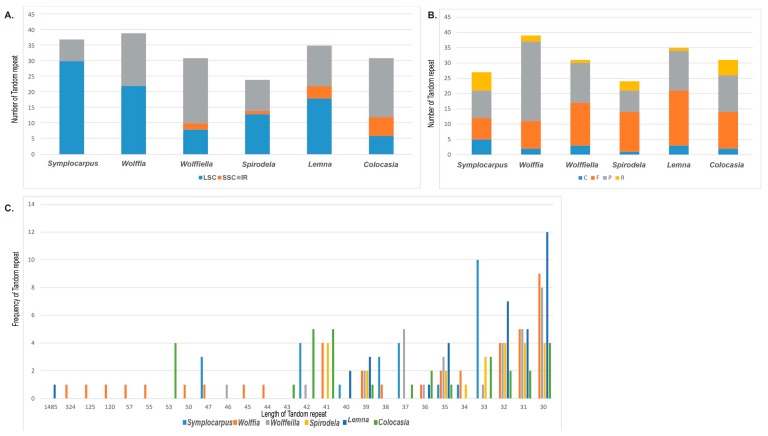
Analyses of tandem repeated sequences in the Araceae chloroplast genome. (**A**) Distribution of tandem repeats in Araceae species. F, P, R and C indicate the repeats matching in forward, palindrome, reverse and complement orientation, respectively (**B**) Type of tandem repeats in Araceae species. (**C**) Frequency of tandem repeats sequences in Araceae species.

Four classes of tandem repeats (forward repeats, reverse repeats, complement repeats and palindromic repeats) were found in six Araceae species (*Lemna*, *Spirodela*, *Dieffenbachia*, *Wolffiella*, *Colocasia* and *Symplocarpus*). The number of tandem repeats ranged from 24 to 39 in the six Araceae species, with *Wolffia* showing the highest number of tandem repeats. The tandem repeats in *Symplocarpus*, *Wolffia*, *Spirodela* and *Lemna* were located in the LSC region more often than in the SSC or IR regions. However, larger number of tandem repeats in *Wolffiella* and *Colocasia* were located in the IR region (Figure 4A). *Symplocarpus* contained five complement repeats, seven forward repeats, nine palindromic repeats and six reverse repeats. For the type of SSRs, forward repeats had the highest numbers in *Wolffiella*, *Spirodela*, *Lemna* and *Colocasia* (Table S2). The SSRs in *Wolffia* and *Symplocarpus* had the highest number of palindromic repeats, especially those in *Wolffia*, with 29 palindromic repeats (Figure 4B). The tandem repeats ranged from 30 to 1485 bp and the sizes of most of the tandem repeats were between 30 and 40 bp (Figure 4C).

**Figure 5:**
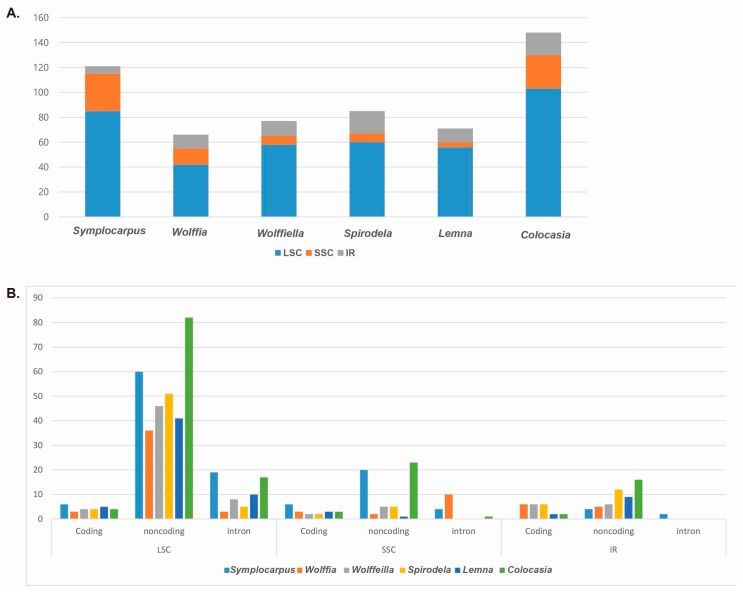
Analyses of simple-sequence repeats (SSRs) in the Araceae chloroplast genome. (**A**) Location of SSRs in Araceae species; (**B**) Distribution and region of SSRs in Araceae species.

Simple sequence repeats are effective markers for population genetics. A total of 121 SSRs were present in the *Symplocarpus* cp genome, in addition to 83 mononucleotides, 40 dinucleotides, nine trinucleotides, 13 tetranucleotides, one pentanucleotide and one decanucleotide repeat (Table S3). A total of 85 SSRs were distributed in the LSC region (six in the coding regions, 60 in the noncoding regions and 19 in the introns), whereas the SSC and IR regions had 30 (six in the coding regions, 20 in the noncoding regions and four in the introns) and six (four in non-coding regions and two introns) SSRs, respectively (Figure 5A,B). The total number of SSRs in the genomes of other Araceae species was 66 in *Wolffia*, 77 in *Wolffiella*, 85 in *Spirodela*, 71 in *Lemna* and 148 in *Colocasia* (Figure 5A and Table S3). Most of the SSRs were located in the non-coding regions in the LSC region (Figure 5B).

### 3.4. Two Types of Inverted Repeat Regions in Araceae

**Figure 6:**
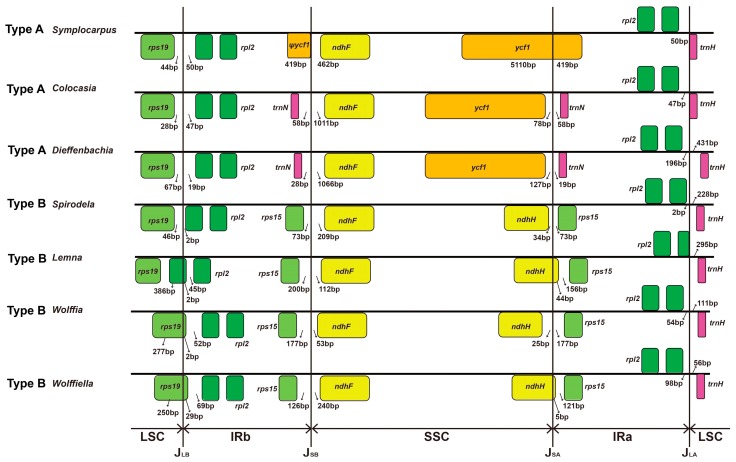
Comparison of LSC, SSC and IR junction positions among Araceae species.

The LSC/IR_B_/SSC/IR_A_ boundary regions of the *S. renifolius* cp genome were compared to those of the other Araceae genomes and two types of such regions were found (Figure 6). Type A was present in *S. renifolius*, *Dieffenbachia* and *Colocasia* and was found at the border between LSC and IR_B,_ between *rpl2* and *rps19*. The IR_B_ and SSC border occurs between *trnN*-GUU and *ndhF*. The IR_B_/SSC border was located between the *trnN*-GUU and *ycf1* and the IR_B_/LSC border was located between *rpl2* and *trnH*-GUG. The sizes of the IRs in *S. renifolius*, *Dieffenbachia* and *Colocasia* were 25,801, 25,273 and 25,235 bp, respectively (Table 1). Type B occurred in *Lemna*, *Spirodela*, *Wolffia* and *Wolffiella*. These four plants showed expansion of the IRs, such as duplication of *ycf1* and *rps15*. The IR lengths in *Spirodela*, *Lemna*, *Wolffiella* and *Wolffia* were longer than in the four Type A species (31,223, 31,755, 31,683 and 31,930 bp, respectively, Table 1). The borders between the LSC/IR_B_ and IR_A_/LSC were located in the same place as in Type A. However, IR_B_/SSC and SSC/IR_A_ borders were located between *rps15*/*ndhF* and *ndhH*/*rps15*, respectively.

### 3.5. Phylogenetic Analysis of Araceae

**Figure 7:**
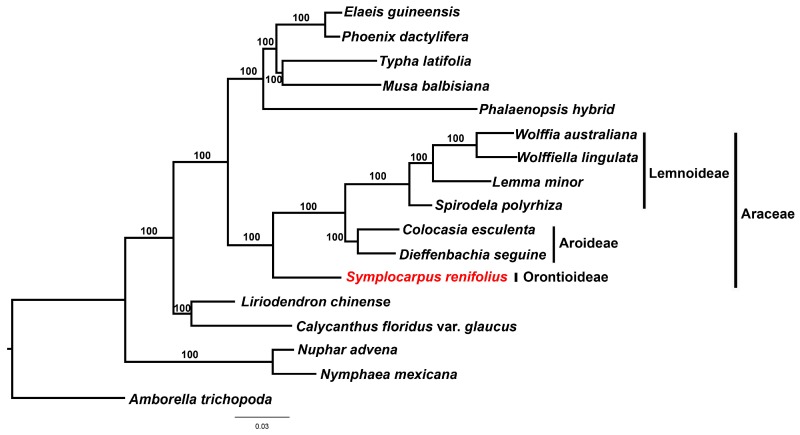
Phylogenetic tree reconstruction of 17 taxa using maximum likelihood method based on concatenated sequences of 77 protein-coding genes. Bootstrap support values > 50% are given at the nodes.

The 77 genes comprising 67,982 bp were used for ML analysis (Figure 7). The ML analysis resulted in a tree with ML values of –lnL = 303,581.50476. Araceae was well supported as monophyletic (100% bootstrap values, BS) and is shown to be a sister family to other monocots with 100% BS. The clades of *Symplocarpus* (Orontioideae), *Colocasia* + *Dieffenbachia* (Aroideae) and *Spirodela* + *Lemna* + *Wolffiella* + *Wolffia* (Lemnoideae) were supported with 100% bootstrap values, respectively. Lemnoideae (*Spirodela*, *Lemna*, *Wolffiella* and *Wolffia*) and other Aroideae species (*Colocasia* and *Dieffenbachia*) formed after *Symplocarpus* in the ML tree.

## 4. Discussion

### 4.1. Gene Loss of infA and Gene Duplication of psbZ in Araceae

The translation initiation factor 1, the product of *infA*, has been lost from many angiosperms and *infA* genes of some plants were transferred to the nucleus [29,30,31]. Studies on *infA* in angiosperms have indicated that it has been independently lost multiple times [29]. The data collected by Ahmed et al. [21] indicate that *infA* is completely missing in duckweed (*Spirodela*, *Lemna*, *Wolffiella* and *Wolffia*), taro (*Colocasia*) and *Dieffenbachia* (NC_27272), with internal stop codons in *infA*. However, *Symplocarpus* contains *infA* (Figure 2). *infA* was lost from Aroideae (*Colocasia* and *Dieffenbachia*) and Lemnoideae (*Spirodela*, *Lemna*, *Wolffiella* and *Wolffia*) and from the completely sequenced Orontioideae subfamily (*Symplocarpus*). Consequently, this result suggests the loss or pseudogenization of *infA* after the divergence of Aroideae and Lemnoideae (Figure 7).

The *psb* genes (*psbA*, *B*, *C*, *D*, *E*, *F*, *H*, *I*, *J*, *K*, *L*, *M*, *N*, *T* and *Z*) of the cp genomes encode a subunit of photosystems II [13,32]. Swiatek et al. [33] proposed that the psbZ protein controls the interaction of PSII cores with the light-harvesting antenna. In particular, they suggested that the PSII-LHCII super complexes could no longer be isolated from PsbZ-deficient tobacco plants. In addition, Nelson and Yocum [34] reported that the products of *psbN* and *psbZ* interact with chlorophyll-bound subunits of *psbC* that reach into the thylakoid lumen. Previous studies on *Cuscuta* [35], *Aneura* [36] and *Epifagus* [37] showed the loss of some *psb* genes.

*psbZ* of Araceae plants is located between *trnS*-UGA and *trnG*-UCC in the LSC region (Figure 3). However, *psbZ* in *Wolffia* (JN160605) had a duplication event and was identified at two locations in the LSC region (*trnS-*UGA/*trnG*-UCC and *ycf3*/*trnS*-GGA). Interestingly, seven tandem repeats of *Wolffia* were located in the *trnS*-UGA/*trnG*-UCC and *ycf3*/*trnS*-GGA regions (Figure 3, Table S2) and all the tandem repeats were palindromic. The tandem repeats were not found in other Araceae plants, except in *Wolffia* in the *trnS*-UGA/*trnG*-UCC and *ycf3*/*trnS*-GGA regions. Our results suggest that the palindromic repeats are effective for *psbZ* duplication.

### 4.2. Phylogenetic Analysis and cp Structure in Araceae

**Figure 8:**
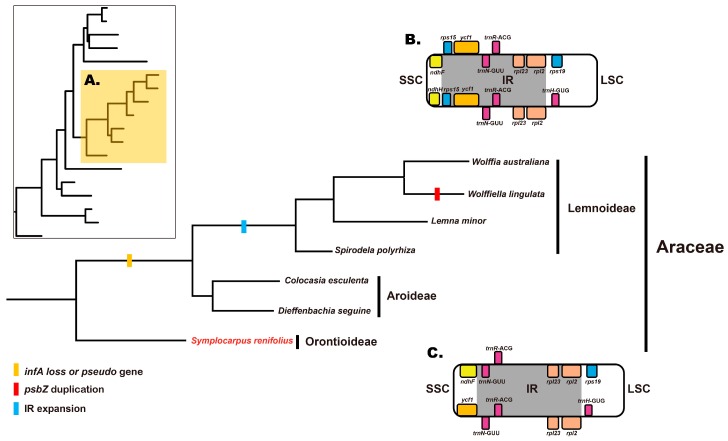
Gene duplication, gene loss and IR expansion in Araceae. (**A**) Phylogenetic position and gene contents in Araceae species; (**B**) IR junction expansion and gene duplication in Lemnoideae; (**C**) IR junction in Aroideae and Orontioideae.

The Araceae family is divided into two groups: the Proto-Araceae (Orontioideae) and the Spirodela clade (Figure 8). The Spirodela clade is composed of Lemnoideae (Duckweeds) and the True Araceae, as determined by molecular phylogenetic analysis (*rbcL*, *matK*, *trnK* intron, *trnL* intron and *trnL*-*trnF* IGS) and morphological data analysis [3,38]. Our results support those of previous studies showing that Araceae are a well-defined group with three groups within it (Figure 8A): Orontioideae (*Symplocarpus*), Aroideae (*Colocasia* and *Dieffenbachia*) and Lemnoideae (*Spirodela*, *Lemna*, *Wolffiella* and *Wolffia*).

Inverted repeat regions are variable sites and useful features for plant [18,39]. The expansion of the IR occurred within a few angiosperm families and groups, such as Eleagnaceae [31], Geraniaceae [16], *Ipomoea* [35], *Oryza* [40] and maize [41].

The species within the Lemnoideae (*Spirodela*, *Lemna*, *Wolffiella* and *Wolffia*) subfamily are different from the other Araceae species. The Lemnoideae species are miniscule in size and are aquatic monocotyledons. In our results (Figure 8), it was interesting to note that Lemnoideae were an independent clade and had different IR gene contents in their cp genomes. In the four species of Lemnoideae, the IR region was ca. 6 kb larger than that of the other Araceae species (Table 1). In Lemnoideae, the IR_B_/SSC and SSC/IR_A_ regions have expanded to include *ycf1* and *rps15* gene duplications (Figure 8B), unlike that in other Araceae species (Figure 8C). This structure supported the theory of independent evolution of the IR regions in the Araceae family.

## Figures and Tables

**Table 1:** General features of *Symplocarpus* and comparison to other Araceae species.

	*Symplocarpus*	*Colocasia*	*Dieffenbachia*	*Lemna*	*Spirodela*	*Wolffiella*	*Wolffia*
Total length	158,521 bp	162,424 bp	163,699 bp	165,955 bp	168,788 bp	169,337 bp	168,704 bp
LSC	86,620 bp	86,670 bp	90,780 bp	89,906 bp	91,222 bp	92,015 bp	91,454 bp
SSC	20,299 bp	22,208 bp	22,440 bp	13,603 bp	14,056 bp	13,956 bp	13,394 bp
IR	25,801 bp	25,273 bp	25,235 bp	31,223 bp	31,755 bp	31,683 bp	31,930 bp
% AT content	62.7%	63.8%	63.6%	64.3%	64.3%	64.2%	64.1%
Genes							
Coding genes	80	79	79	79	79	79	79
tRNA	30	30	30	30	30	30	30
rRNA	4	4	4	4	4	4	4
IR duplication genes	17	17	17	17	19	19	19

LSC: Large Single Copy, SSC: Small Single Copy, IR: Inverted Repeat, tRNA: transfer RNA, rRNA: ribosomal RNA.

## Supplementary Materials

The following are available online at www.mdpi.com/2073-4425/8/11/324/s1. Supplementary Table S1. Taxa used in the phylogenetic analysis and GenBank accession numbers of the references, Supplementary Table S2. Tandem repeat sequence of Araceae, Supplementary Table S3. Simple sequence repeats of Araceae.
