# Supplementary material for: The Chloroplast Genome of Symplocarpus renifolius: A Comparison of Chloroplast Genome Structure in Araceae
Source: Genes (Basel). 2017 Nov 16;8(11):324. doi: 10.3390/genes8110324 (PMC5704237; doi:10.3390/genes8110324)
Supplement: Supplementary file 1 [file genes-08-00324-s001.zip › Supplementary Table S1.docx]

Supplementary table 1. Phylogenetic study taxa and Genbank accession number of references.

| Species | Accession number |
| --- | --- |
| *Amborella trichopoda* | NC_005086 |
| *Nymphaea mexicana* | NC_024542 |
| *Nuphar advena* | NC_008788 |
| *Calycanthus floridus* var. *glaucus* | NC_004993 |
| *Liriodendron chinense* | NC_030504 |
| *Elaeis guineensis* | NC_017602 |
| *Phoenix dactylifera* | NC_013991 |
| *Typha latifolia* | NC_013823 |
| *Musa balbisiana* | NC_028439 |
| *Phalaenopsis hybrid* | NC_025593 |
| *Wolffia australiana* | JN160605 |
| *Wolffiella lingulata* | JN160604 |
| *Lemna minor* | NC_010109 |
| *Spirodela polyrhiza* | JN160603 |
| *Colocasia esculenta* | JN105690 |
| *Diffenbachia seguine* | NC_027272 |
| *Symplocarpus renifolius* | KY039276 |

* 77 genes: *atpA, atpB, atpE, atpF, atpH, ccsA, cemA, clpP, infA, matK, ndhA, ndhB, ndhC, ndhD, ndhE, ndhF, ndhG, ndhH, ndhI, ndhJ, ndhK, petA, petB, petD, petG, petL, petN, psaA, psaB, psaC, psaI, psaJ, psbA, psbB, psbC, psbD, psbE, psbF, psbH, psbI, psbJ, psbK, psbL, psbM, psbN, psbT, psbZ, rbcL, rpl2, rpl14, rpl16, rpl20, rpl22, rpl23, rpl32, rpl33, rpl36, rpoA, rpoB, rpoC1, rpoC2, rps2, rps3, rps4, rps7, rps8, rps11, rps14, rps15, rps16, rps18, rps19, ycf1, ycf2, ycf3, ycf4*
